# Author Correction: A human immune dysregulation syndrome characterized by severe hyperinflammation with a homozygous nonsense Roquin-1 mutation

**DOI:** 10.1038/s41467-019-13379-9

**Published:** 2019-11-20

**Authors:** S. J. Tavernier, V. Athanasopoulos, P. Verloo, G. Behrens, J. Staal, D. J. Bogaert, L. Naesens, M. De Bruyne, S. Van Gassen, E. Parthoens, J. Ellyard, J. Cappello, L. X. Morris, H. Van Gorp, G. Van Isterdael, Y. Saeys, M. Lamkanfi, P. Schelstraete, J. Dehoorne, V. Bordon, R. Van Coster, B. N. Lambrecht, B. Menten, R. Beyaert, C. G. Vinuesa, V. Heissmeyer, M. Dullaers, F. Haerynck

**Affiliations:** 1 Primary Immune Deficiency Research Lab, Department of Internal Medicine and Pediatrics, Centre for Primary Immunodeficiency Ghent, Jeffrey Modell Diagnosis and Research Centre, Ghent University Hospital, Ghent, Belgium; 2 VIB Center for Inflammation Research, Unit of Molecular Signal Transduction in Inflammation, Ghent, Belgium; 3 Department of Biomedical Molecular Biology, Ghent University, Ghent, Belgium; 4 Department of Immunology and Infectious Disease and Center for Personalised Immunology (NHMRC Centre for Research Excellence), John Curtin School of Medical Research, Australian National University, Canberra, Australia; 5 Centre for Personalised Immunology (CACPI), Shanghai Renji Hospital, Shanghai Jiao Tong University, Shanghai, China; 6 Department of Internal Medicine and Pediatrics, Division of Pediatric Neurology and Metabolism, Ghent University Hospital, Ghent, Belgium; 7 Institute for Immunology, Biomedical Center, Ludwig-Maximilians-Universität München, Planegg-Martinsried, Germany; 8 Research Unit Molecular Immune Regulation, Helmholtz Zentrum München, Munich, Germany; 9 Department of Internal Medicine and Pediatrics, Division of Pediatric Immunology and Pulmonology, Ghent University Hospital, Ghent, Belgium; 10 Department of Internal Medicine and Pediatrics, Ghent University Hospital, Ghent, Belgium; 11 Center for Medical Genetics, Ghent University Hospital, Ghent, Belgium; 12 VIB Center for Inflammation Research, Unit of Data Mining and Modeling for Biomedicine, Ghent, Belgium; 13 Department of Applied Mathematics, Computer Science and Statistics, Ghent University, Gent, Belgium; 14 VIB Bioimaging Core, VIB Center for Inflammation Research, Ghent, Belgium; 15 The Australian Phenomics Facility, John Curtin School of Medical Research, Australian National University, Canberra, Australia; 16 VIB Center for Inflammation Research, Ghent, Belgium; 17 VIB Flow Core, VIB Center for Inflammation Research, Ghent, Belgium; 18 Department of Internal Medicine and Pediatrics, Division of Pediatric Rheumatology, Ghent University Hospital, Ghent, Belgium; 19 Department of Internal Medicine and Pediatrics, Division of Pulmonology, Ghent University Hospital, Ghent, Belgium; 20 VIB Center for Inflammation Research, Unit for Immunoregulation and Mucosal Immunology, Ghent, Belgium; 21 Department of Pulmonary Medicine, ErasmusMC, Rotterdam, The Netherlands; 22 Ablynx, a Sanofi Company, Zwijnaarde, Belgium

**Keywords:** Disease genetics, Inflammation, RNA decay, Primary immunodeficiency disorders

Correction to: *Nature Communications* 10.1038/s41467-019-12704-6, published online 21 October 2019

The original version of the Supplementary Information associated with this Article included an incorrect Supplementary Information file, in which only the first page of the file was included. The HTML has been updated to include a corrected and complete version of the Supplementary Information file.

